# A new species of *Scaphosepalum* (Orchidaceae, Pleurothallidinae) from eastern Ecuador

**DOI:** 10.3897/BDJ.14.e176579

**Published:** 2026-03-17

**Authors:** Nadia Lapo-Gonzalez, Gabriel A. Iturralde, Johny J. Mendoza Uyaguari, Jefferson Medina, J. R. Kuethe, Henry X. Garzón-Suárez, Luis E. Baquero, Marco M. Jiménez

**Affiliations:** 1 Floplaya, Compañía de flores y plantas Yantzaza S.A., El Pangui, Ecuador Floplaya, Compañía de flores y plantas Yantzaza S.A. El Pangui Ecuador; 2 Grupo Científico Calaway Dodson: Investigación y Conservación de Orquídeas del Ecuador, Quito, Ecuador Grupo Científico Calaway Dodson: Investigación y Conservación de Orquídeas del Ecuador Quito Ecuador; 3 Grupo de Investigación en Biodiversidad, Medio Ambiente y Salud (BIOMAS), Carrera de Ingeniería Agroindustrial, Facultad de Ingenierías y Ciencias Aplicadas, Universidad de Las Américas (UDLA), Vía a Nayón, Quito, Ecuador Grupo de Investigación en Biodiversidad, Medio Ambiente y Salud (BIOMAS), Carrera de Ingeniería Agroindustrial, Facultad de Ingenierías y Ciencias Aplicadas, Universidad de Las Américas (UDLA), Vía a Nayón Quito Ecuador; 4 Amichi Lounge, Vía a Gualaquiza - San Juan Bosco, E45 s/n., Gualaquiza, Ecuador Amichi Lounge, Vía a Gualaquiza - San Juan Bosco, E45 s/n. Gualaquiza Ecuador; 5 Grupo de Investigación Biodiversidad de Ecosistemas Tropicales (BIETROP), Universidad Técnica Particular de Loja, Loja, Ecuador Grupo de Investigación Biodiversidad de Ecosistemas Tropicales (BIETROP), Universidad Técnica Particular de Loja Loja Ecuador; 6 School of Environment, Earth and Life Sciences campus, Symonds Street 1010,University of Auckland, Auckland, New Zealand School of Environment, Earth and Life Sciences campus, Symonds Street 1010,University of Auckland Auckland New Zealand; 7 Herbario HUTPL, Departamento de Ciencias Biológicas, Universidad Técnica Particular de Loja, Loja, Ecuador Herbario HUTPL, Departamento de Ciencias Biológicas, Universidad Técnica Particular de Loja Loja Ecuador; 8 Jungle Dave’s Science Foundation, San Juan Bosco, Ecuador Jungle Dave’s Science Foundation San Juan Bosco Ecuador

**Keywords:** Cordillera del Cóndor, rainforest, *Scaphosepalum lesterlapoi*, *Scaphosepalum pleurothallodes*, south-eastern Ecuador

## Abstract

**Background:**

The genus *Scaphosepalum* comprises a group of epiphytic orchids, distinguished by their non-resupinate flowers and prominent osmophores located on the distal portion of the synsepal. With over 60 recognised species, *Scaphosepalum* is distributed throughout the tropical Americas, with its highest diversity in Colombia and Ecuador. Recent explorations in the Eastern Andes and the Cordillera del Cóndor in south-eastern Ecuador led to the discovery of several new orchid species, including an endemic *Scaphosepalum* taxon. Many regions remain underexplored, leaving the orchid flora to continually reveal previously undocumented diversity.

**New information:**

Herein, we describe and illustrate *Scaphosepalum lesterlapoi*, as new species discovered in the Andean-Amazonian piedmont of eastern Ecuador. This taxon was initially misidentified as *S. medinae* based on photographic records. It is compared with *S. pleurothallodes* and *S. medinae*, from which it is distinguished by its maroon to yellow-maroon flowers and the rhombic-spathulate shape of the petals. This denotes a marked difference to the yellow flowers spotted with red and the obovate petals of *S. pleurothallodes* or the lavender with white cells and light brown flowers and the narrowly obtuse petals of *S. medinae*. The new species is currently known from three localities in the Quimi and Talag River basins. Due to its restricted distribution, small population size and threats from cattle grazing and mining activities, we propose its classification as Critically Endangered under the B criterion of the IUCN.

## Introduction

The orchid genus *Scaphosepalum* Pfitzer was established in 1888 to accommodate species formerly placed in *Masdevallia* Ruiz & Pav., including *Masdevallia verrucosa* Rchb.f. and *M. ochthodes* Rchb.f. In 1890, Rolfe transferred the remaining species that were known at that time as *Masdevallia*; these species are now considered part of *Scaphosepalum* (Rolfe 1890). Kraenzlin already recognised it as an orchid genus in 1925 (Kraenzlin 1925). Since the mid-1980s, it has been accepted by Luer (1988) and Pridgeon (2005). Species of the genus are characterised by non-resupinated flowers with well-developed osmophores, ending with caudae, in the distal portion of the synsepal (Luer 1988, Pridgeon 2005). Molecular studies on Pleurothallidinae suggest that *Scaphosepalum* is likely monophyletic — although additional sampling is needed to confirm this — and it is closely related to *Platystele* Schltr. and *Teagueia (Luer) Luer*, within a broader clade that also includes *Specklinia* Lindl., *Dryadella* Luer, *Andreettaea* Luer and *Andinia* (Luer) Luer (Endara A. et al. 2011, Karremans 2016, Trávníček et al. 2025).

Karremans and Vieira-Uribe (2020) account for 54 species of *Scaphosepalum*, which are distributed across the tropical Americas from Mexico to Bolivia and the Guianan Shield (Luer 1988, Karremans 2016, Karremans et al. 2016). Six of these species have been discovered within the last five years, increasing the total number of species for the genus to 60 (Vierling 2020, Jiménez et al. 2021, Pupulin and collaborators 2022, Vierling 2022, Vierling 2023). The northern Andes harbour the greatest richness of species diversity (Endara A. et al. 2011), with, in particular, Colombia and Ecuador representing the centre of species diversity for *Scaphosepalum* (Karremans et al. 2016).

The Eastern Andes and the Cordillera del Cóndor in south-eastern Ecuador form part of the Tropical Andes biodiversity hotspot (Gerique 2011, Chicaiza and Yánez 2013), where recent botanical expeditions over the past five years have significantly increased the number of documented orchid species (e.g. Doucette et al. (2023a), Doucette et al. (2023b), Jiménez et al. (2023), Jiménez et al. (2024), Lapo-Gonzalez et al. (2024), Vélez-Abarca et al. (2024)). Within this region, twelve species of *Scaphosepalum* have been recorded: the widely distributed *S. antenniferum* Rolfe and six endemics — *S. andreettae* Luer, *S. aociorum* M.M. Jiménez, Baquero & Vélez-Abarca, *S. dalstroemii* Luer, *S. globosum* Luer & Hirtz, *S. merinoi* Luer and *S. triceratops* Luer & Andreetta (Luer 1988, Luer 2002, Jiménez et al. 2021). Additionally, five further species were identified through herbarium collections: *S. breve* (Rchb.f.) Rolfe, *S. ovulare* Luer, *S. pulvinare* Rchb.f. & Rolfe, *S. rapax* Luer and *S. tiaratum* Luer, which are cited below under specimens examined.

A new species morphologically similar to *Scaphosepalum pleurothallodes* Luer & Hirtz was first documented photographically (without a herbarium voucher), but misidentified as *S. medinae* Luer & Portilla (Dodson 2004). This same species was recently found again in south-eastern Ecuador and studied in more detail, leading to the conclusion that it belongs to a new species. In this paper, we formally describe *Scaphosepalum lesterlapoi* as a new species, along with a detailed comparison with its nearest related species, information about its distribution and habitat, proposed conservation status and illustrations depicting the species.

## Materials and methods

The material used to describe and illustrate the new species was collected during a field expedition in 2023, as part of two research projects entitled “Diversidad de orquídeas terrestres y epífitas en dos tipos de bosque del cantón El Pangui, provincia de Zamora Chinchipe” and “Estudio de Diversidad Taxonómica y Genética de Plantas y Hongos del Ecuador”. These projects were conducted under permits issued by the Ministerio del Ambiente del Ecuador. High-definition digital photographs were taken using a Panasonic Lumix FZ300 camera, equipped with Raynox DCR-250 (50 mm) and MSN-505 (37 mm) super macro lenses. The full anatomy of the plant was photographed against black chamois fabric backgrounds, complete with reference scale, allowing accurate morphological measurements to be taken directly from the fresh material using ImageJ's open-source software (González 2018). A Lankester Composite Dissection Plate (LCDP) was prepared in Adobe Photoshop CS6 Portable 2020, following the recommendations of Karremans et al. (2024).

To compare the new species with other congeners, we reviewed the taxonomic treatment of *Scaphosepalum* (Luer 1988), examined descriptions of similar taxa published subsequently (Luer 1992, Luer 2000, Baquero R. 2017, Baquero et al. 2018, Vierling 2018, Baquero 2019, Vierling 2020, Jiménez et al. 2021, Pupulin and collaborators 2022, Vierling 2022, Vierling 2023) and consulted available illustrations. All sources were accessed through digital libraries. All the specimens identified as *Scaphosepalum* from south-eastern Ecuador and stored at MO, QCA and SEL herbaria (Thiers 2025) were examined online through Data Web Ecuador (https://bioweb.bio/portal/), IDigBio Portal (https://portal.idigbio.org/portal/search) and Tropicos (https://tropicos.org/home). All specimens of *Scaphosepalum* deposited at the LOJA and HUTPL Herbaria were evaluated and consulted. The extent of occurrence (EOO) and the area of occupancy (AOO) were calculated using the GeoCAT tool endorsed by RBG Kew (Bachman et al. 2011) to assess the conservation status of the species under the geographical range or B criterion of the IUCN (IUCN 2024) and complement the other IUCN criteria as outlined by this institution. The distribution map was prepared using ArcGIS Desktop 10.8 (ESRI 2020).

## Taxon treatments

### Scaphosepalum lesterlapoi

N. Lapo-Gonzalez & M.M.Jiménez sp. nov.

A9EC35E0-C8C5-5C11-8F06-70729E1AA598

77377796-1

#### Materials

- **Type status:** Holotype. **Occurrence:** catalogNumber: LOJA 42884; recordNumber: N.Lapo 74; recordedBy: N. Lapo; occurrenceID: 9D5FC121-52C6-53E5-B22C-598958C71FA4; **Taxon:** scientificName: Scaphosepalum lesterlapoi N. Lapo-Gonzalez & M.M.Jiménez; **Location:** country: Ecuador; stateProvince: Morona Santiago; locality: Valle del Quimi; verbatimElevation: 1157 m; **Event:** year: 2023; month: 03; day: 11; **Record Level:** institutionCode: LOJA!
- **Type status:** Paratype. **Occurrence:** catalogNumber: HUTPL 15572; recordNumber: M.M Jiménez 1937; recordedBy: M.M. Jiménez; occurrenceID: 6CF2B5A0-415C-586C-A7B0-C41763DA2569; **Location:** country: Ecuador; stateProvince: Zamora Chinchipe; locality: Cerca de Tundayme, por un río; verbatimElevation: 989 m; verbatimLatitude: 17 M 778832.30 m E; verbatimLongitude: 9605337.94 m S; **Event:** year: 2023; month: 10; day: 30
- **Type status:** Paratype. **Occurrence:** recordNumber: A. Hirtz 8237; recordedBy: A. Hirtz; occurrenceID: D8A6033B-C299-54E4-BED6-524B36C6EC25; **Location:** country: Ecuador; stateProvince: Napo; locality: Shandia, S of Tena; verbatimElevation: 800 m; verbatimCoordinates: 18 M 196594.66 m E; verbatimLatitude: 9890640.06 m S; **Event:** year: 2002; month: 07; day: 2; **Material Entity:** materialEntityType: Photographic

#### Description

**Figure 1.**
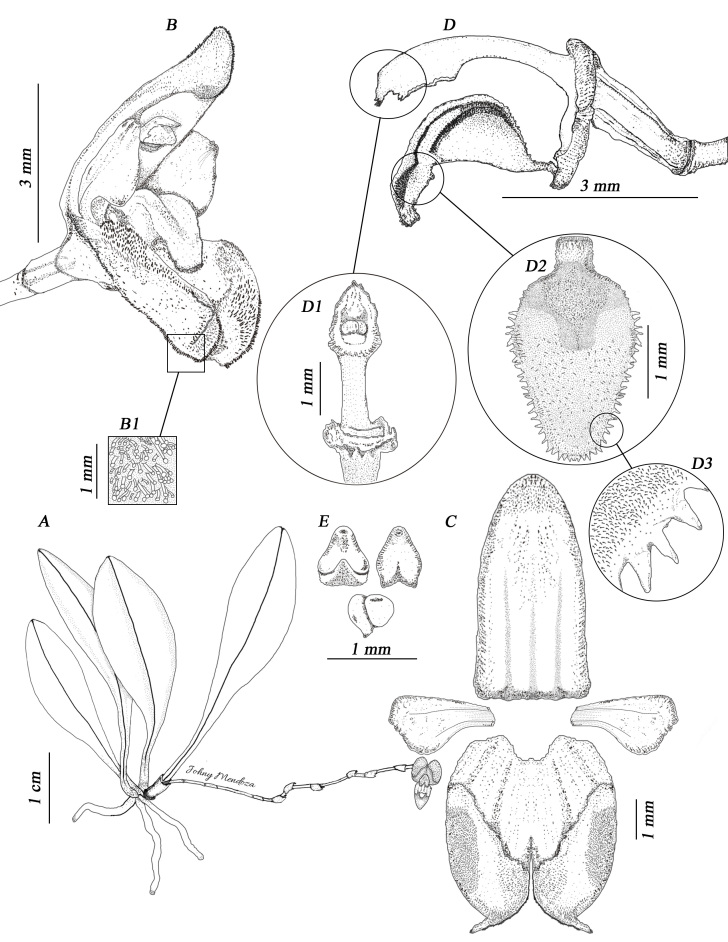
*Scaphosepalum lesterlapoi* N. Lapo-Gonzalez & M.M.Jiménez. **A** Habit; **B** Flower; **B1** Close-up of the osmophore; **C** Dissected perianth; **D** Column, ovary and lip, lateral view; **D1** Column, ventral view; **D2** Lip, adaxial view; **D3** Close-up of the edge of the lip; **E** Anther and pollinia. Illustration by Johny J. Mendoza Uyaguari, based on the holotype.

**Figure 2.**
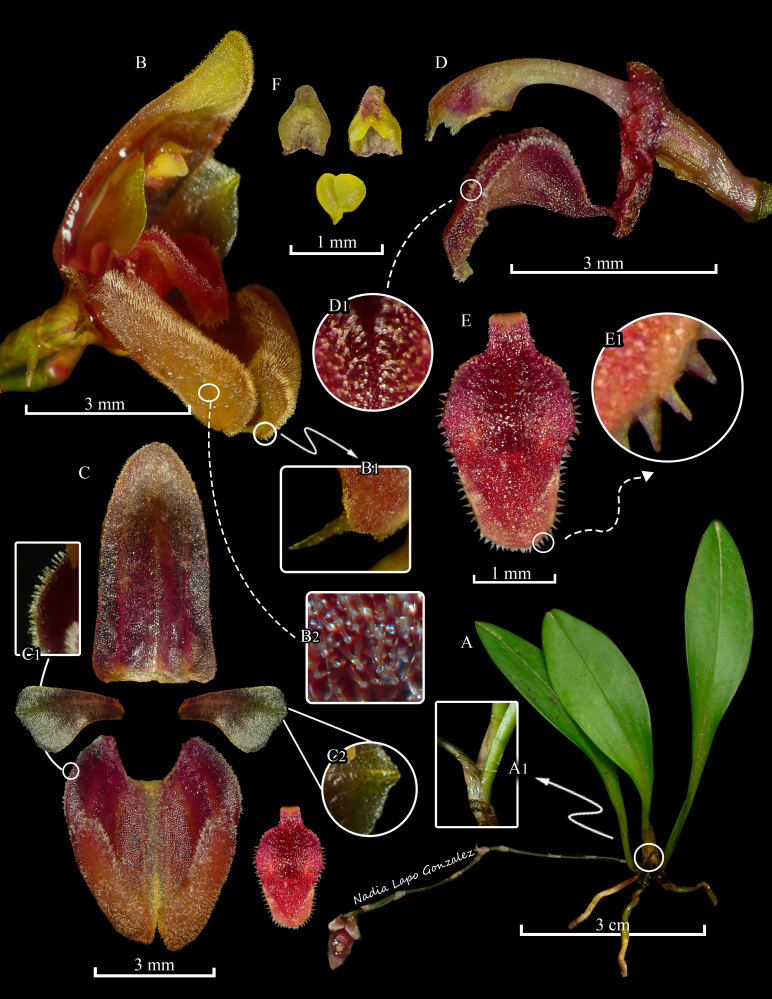
*Scaphosepalum lesterlapoi* N. Lapo-Gonzalez & M.M.Jiménez. **A** Habit; **A1** Close-up of the junction between the ramicaul and the inflorescence; **B** Flower; **B1** Close-up of the tails of the synsepal; **B2** Close-up of the osmophore; **C** Dissected perianth; **C1** Close-up of the basal margin of the synsepal; **C2** Close-up the petal apex; **D** Column, ovary and lip, lateral view; **D1** Close-up of the lip adaxial surface; **E** Lip, adaxial view; **E1** Close-up of the margin the lip; **F** Anther and pollinia. Plate by N. Lapo-Gonzalez, based on photographs of the holotype taken by M.M. Jiménez.

*Herb* epiphytic, caespitose, to 7.5 cm tall, including the inflorescence. *Roots* slender, 0.4–0.7 mm in diameter, thin, light brown. *Ramicauls* erect to sub-erect, 4.0–6.0 mm long, enclosed by 1 sheath. *Leaf* 2.6–5.5 × 0.8–1.0 cm, erect, elliptic-oblanceolate, including the petiole, base abruptly attenuate, margin entire, apex acute; petiole slender, channelled. *Inflorescence* a loose or rarely congested, flexuous, successively flowered raceme up to 5.2–7.0 cm long, borne by a slender, sub-horizontal peduncle, 4.1–5.7 cm, arising from the base of the ramicaul; floral bracts thin, obliquely funnel-shape, 1.7 mm long, acute; pedicel 2–3 mm long; ovary 2.2 × 0.9 mm, 3-ribbed, pale greenish-red, obconic, papillose. *Flowers* 7.9–8.6 × 3.8–4.1 mm, sepals maroon to yellow with yellow or red osmophores, petals reddish at the base, becoming yellow to the apex, lip maroon. *Dorsal sepal* 5.0–6.0 × 2.4–2.7 mm, oblong-lanceolate, obtuse, tricarinate, concave, 3-veined, margin ciliate to the base, the apical third convex, recurved, apex slightly thickened, covered by glandular trichomes. *Lateral sepals* 4.7–5.2 × 3.7–4.2 mm, connate for 3 mm into a 6–veined, bifid, concave, broadly elliptic synsepal, 4-carinate abaxially, the distal margin of each lateral sepal occupied by a thick, oblique, oblong-pandurate, 4.0 × 1.1–1.3 mm, densely pubescent osmophore, margin revolute, ciliate to the base, trichomes glandular; apices obtuse, each one contracted into a slender, incurved, very short, 0.8 mm long, verrucose, yellow or reddish tail. *Petals* 2.4–2.7 × 1.3–1.7 mm, 2-veined, obliquely, rhombic-spathulate, glabrous, verrucose, margin entire, provided with two longitudinal ribs abaxially; apex broadly obtuse and shortly apiculate. *Lip* 2.4–3 × 1.4–1.6 mm, clawed, arching and conduplicate in natural position; claw strap-shaped and quadrangular; blade obovate-sagittate, rounded, convex and laciniate before the middle to the apex, with erect, obtuse margins in the median portion, trichomes densely distributed, microscopically glandular, increasing in length from the middle towards the margin. *Column* 2.3–3.0 mm long, yellowish-green to red, semi-terete, arcuate, winged above the middle, clinandrium 3-denticulate, rostellum semi-lunate, foot 1.3 mm long, glandular. *Anther* 0.8–1.0 × 0.6 mm, cucullate, yellowish-green with a touch of purple-red at the endings, microscopically verrucose. *Pollinia* 2, 0.5 × 0.3 mm, broadly obovoid, yellow (Fig. 1, Fig. 2).

#### Diagnosis

This species is similar to *Scaphosepalum pleurothallodes* Luer & Hirtz, but it differs by having a sub-horizontal inflorescence (vs. erect), maroon to red flowers with yellow or red osmophores that are longer and narrower, 3.8 × 1.3 mm (vs. yellow flowers with red spots, with shorter and thicker osmophores, 3.0 × 1.5 mm), petals rhombic-spathulate, apex broadly obtuse and shortly apiculate (vs. obovate petals with acute and 3-toothed in the apex) and a lip with a clawed, obovate-sagittate, rounded, covered by papillose trichomes increasing in length from the middle towards the margin (vs. truncate at the base, obovate-pandurate and pubescent throughout).

#### Etymology

The specific epithet honours Lester Lapo, an outstanding orchid grower from El Pangui, Zamora-Chinchipe Province, southern Ecuador and who first discovered this species.

#### Distribution

**Figure 3.**
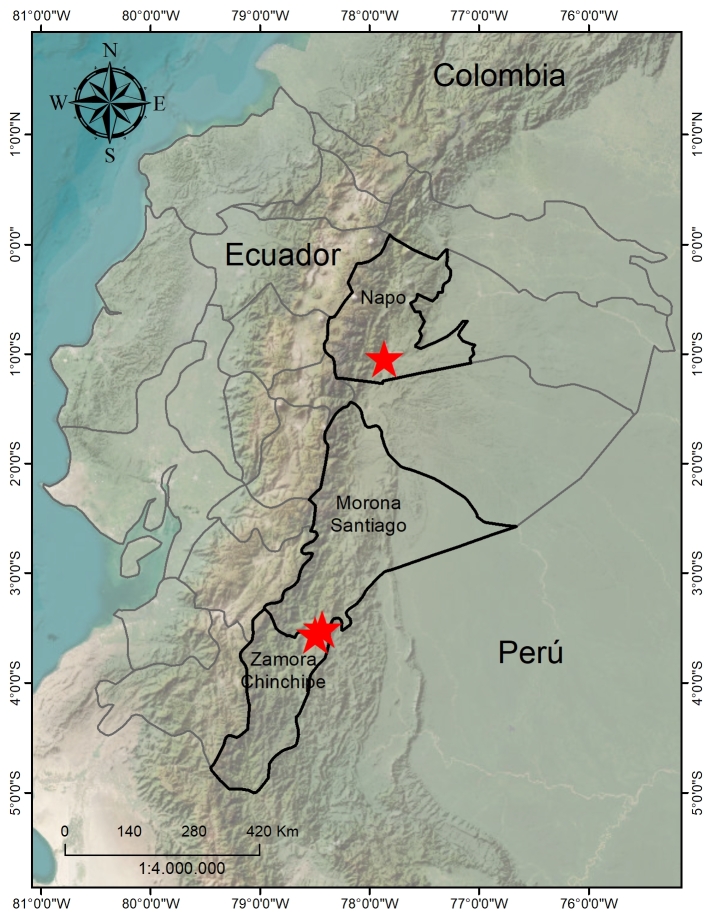
Distribution map of *Scaphosepalum lesterlapoi*. Produced by Henry X. Garzón-Suárez.

*Scaphosepalum lesterlapoi* is currently known from three localities in eastern Ecuador. The first, the type locality is in Valle del Quimi, at an altitude of 1157 m. Individuals have been observed growing on tree trunks in a premontane forest, between the provinces of Zamora-Chinchipe and Morona-Santiago, in the Cordillera del Cóndor region. A few metres from this locality, pastureland and mining activity were observed. The second locality is located 9.5 km south of the type locality. Here, individuals of the new species were observed for the first time in 2018 and 2019, growing at an altitude of 989 m, in a forest disturbed by deforestation and mining activities near Tundayme. The third locality is located in Shandia, near the town of Tena (Napo Province), where a third specimen was photographically documented, but was erroneously identified as *Scaphosepalum medinae* by Dodson (2004). It corresponds to the collection of *Hirtz et al. 8237*, thus extending the distribution of the species to north-eastern Ecuador (Fig. 3).

#### Ecology

It grows epiphytically on tree trunks approximately 2 m above ground, in aggregates of 1–5 individuals per phorophyte, at an altitude 800–1200 m. Associated tree species include *Ficus* sp., *Grias peruviana* Miers, *Gustavia macarenensis* Philipson, *Inga edulis* Mart., *Inga spectabilis* (Vahl) Willd., *Iriartea deltoidea* Ruiz & Pav., *Pouteria caimito* (Ruiz & Pav.) Radlk. and *Psidium guajava* L. In the type locality, the species shares habitat with other orchid species, such as *Mormolyca polyphylla* Garay & Wirth, *Ornithocephalus kruegeri* Rchb.f., *Pleurothallis* sp., *Restrepia condorensis* Luer & Escobar, and *Stelis* sp.

In the second locality, two individuals were found, one co-occurring with *Gongora scaphephorus* Rchb.f. & Warsz., *Masdevallia guerrieroi* Luer & Andreetta and *M. ampullacea* Luer & Andreetta. Commonly observed tree species in this locality were *Ceiba samauma* (Mart.) K. Schum., *Leonia glycycarpa* Ruiz & Pav., *Pachira aquatica* Aubl. and *Terminalia amazonia* (J.F. Gmel.) Exell. Both specimens were later cultivated at the Orquideario La Paphinia in Zamora, Ecuador.

#### Conservation

*Scaphosepalum lesterlapoi* is currently known from only three localities in the Andean–Amazonian piedmont of eastern and south-eastern Ecuador: two within the Quimi River Basin (Morona-Santiago and Zamora Chinchipe Provinces) and one in the Talag River Basin (Napo Province). The species has an estimated extent of occurrence (EOO) of approximately 0.79 km² and an area of occupancy (AOO) of almost 12 km² (calculated using a 2 × 2 km grid), qualifying it as Endangered (EN) under IUCN criterion B1ab(iii)+2ab(iii) (IUCN 2024). Populations are small, scarce and scattered, with a strong dependence on a single valley, making them vulnerable to habitat loss caused by cattle grazing and mining activities. Considering its restricted range, absence from any formally protected area and evident pressure from nearby mining operations, this assessment is considered accurate. Although the species may potentially occur within the adjacent El Quimi Biological Reserve, no specimens have yet been recorded within its boundaries.

#### Taxon discussion

*Scaphosepalum lesterlapoi* belongs to section Leiocalium Luer (Luer 1988), whose members are characterised by slender, glabrous peduncles bearing loose to congested, occasionally distichous racemes with inconspicuous floral bracts.

**Figure 4.**
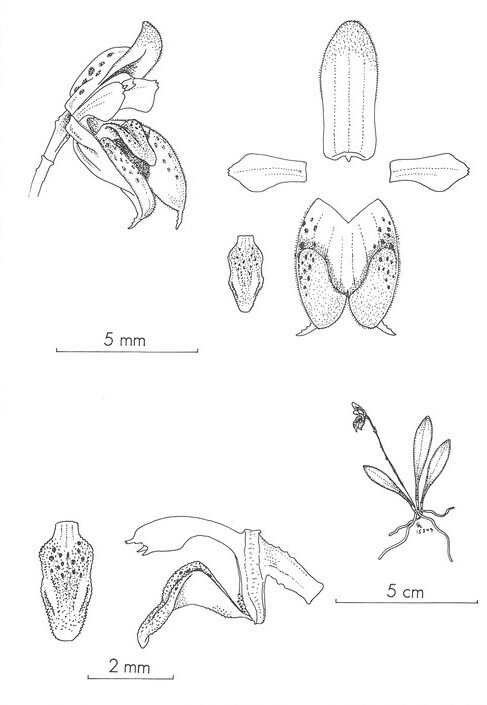
Illustration of *Scaphosepalum pleurothallodes* Luer & Hirtz (from Icones Pleurothallidinarum IX: Systematics of *Myoxanthus* 1992: pl. 48). Courtesy of the Missouri Botanical Gardens Press.

The new species is most similar to *Scaphosepalum pleurothallodes* by sharing the small size of the plants and flowers with oblong dorsal sepals bearing and a synsepal with thickened, pubescent osmophores contracted into very short tails. However, *S. lesterlapoi* differs by its larger habit (up to 7.5 cm vs. 4.5 cm tall), longer leaves (2.6–5.5 cm long vs. 1.5–3.0 cm long), sub-horizontal inflorescence (vs. suberect), flowers maroon to red with, longer and narrower osmophores (vs. flowers yellow with red spots, with shorter and thicker osmophores) and displaying stable colouration patterns in both species. Additionally, the oblong-pandurate osmophore cushions in *Scaphosepalum lesterlapoi* (vs. oblong cushions), the petals with the part facing the lip significantly broader than the part facing the dorsal sepal and the apex broadly obtuse and shortly apiculate (vs. slightly angled, acute and 3-toothed in the apex) (Fig. 4). The lip of *S. lesterlapoi* has an overall different shape compared to other species of the genus, with a blade obovate-sagittate, with a strap-shaped base that is shortly rectangular to quadrangular, but notably conspicuous. The two short, thick, obtuse wings near the base extend along the mid-portion and are covered by glandular trichomes (vs. simple lip with short pubescence), laciniate before the middle to the apex (vs. shortly fimbriate apex) and the lip callose (vs. lip with two low, longitudinal calli near the mid-region).

**Figure 5.**
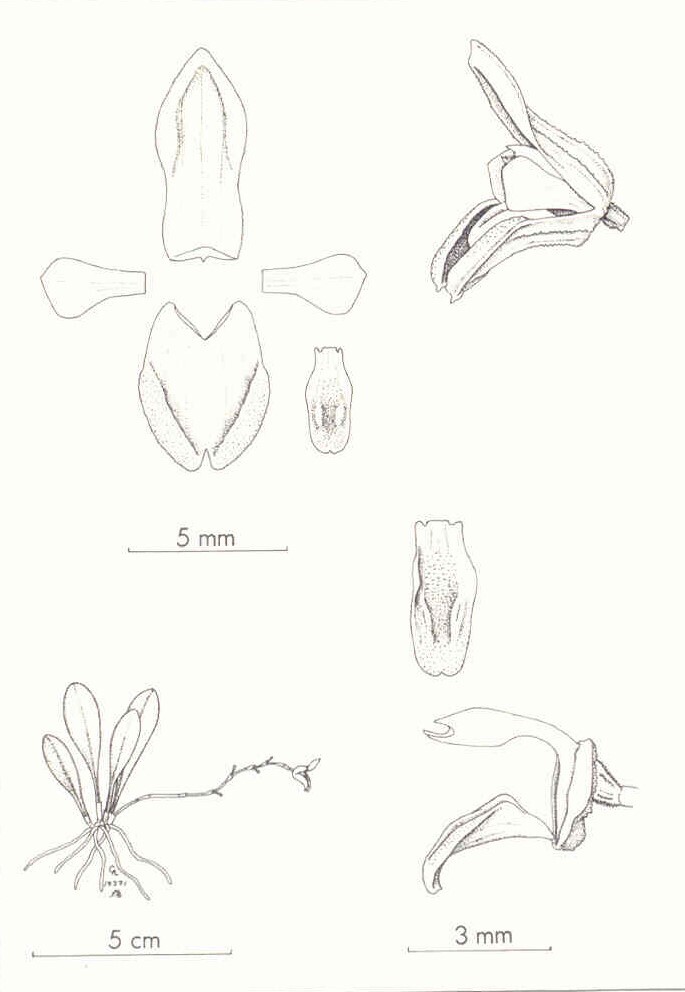
Illustration of *Scaphosepalum medinae* Luer & J.Portilla (from Icones Pleurothallidinarum XX 2000: pl. 139). Courtesy of the Missouri Botanical Gardens Press.

**Figure 6.**
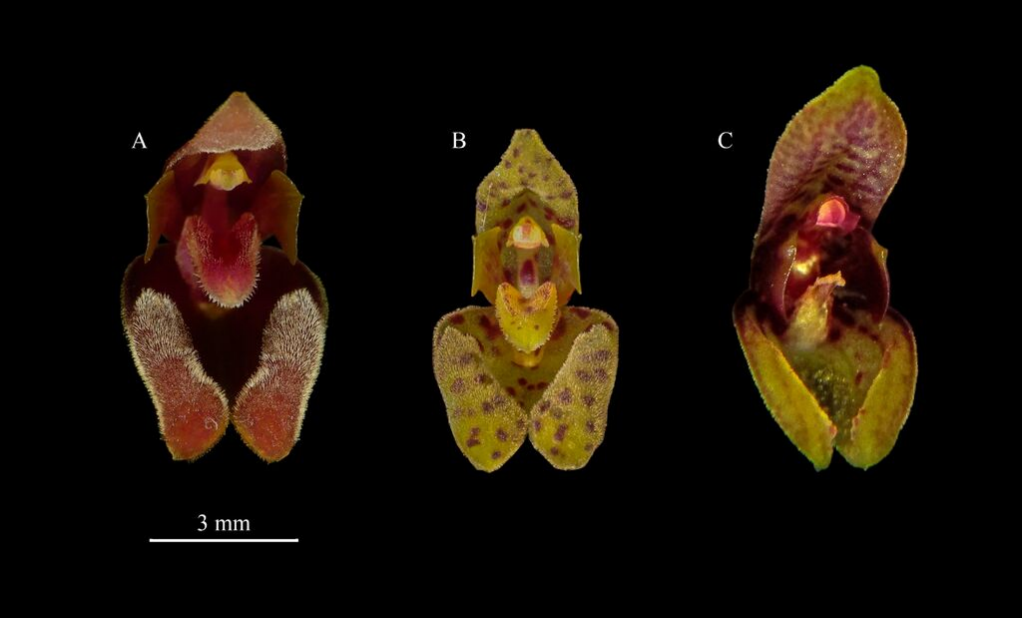
Floral comparison. **A** *Scaphosepalum lesterlapoi* based on M.M. Jiménez 1937; **B** *Scaphosepalum pleurothallodes* by Lourens Grobler; **C** *Scaphosepalum medinae* by Kevin Holcomb. Elaborated by N. Lapo-Gonzalez.

*Scaphosepalum lesterlapoi* is also similar to *S. medinae* Luer & J.Portilla, both sharing oblong sepals and a synsepal with very short tails. However, *Scaphosepalum lesterlapoi* differs by its larger habit (up to 7.5 cm vs. 5.8 cm tall), longer leaves (2.6–5.5 cm vs. 2.0–3.0 cm long), sub-horizontal inflorescence (vs. more or less horizontal), flowers maroon to red with yellow or red osmophores (vs. flowers maroon with yellow and yellow osmophores), oblong-lanceolate dorsal sepal (vs. oblong), the petals obliquely, rhombic-spathulate, apex broadly obtuse and shortly apiculate (vs. obliquely obovate, broadly obtuse, unguiculate), lateral sepals occupied by densely pubescent, oblong-pandurate osmophores along most of their margins (vs. cellular-glandular, longitudinal thickenings just below the middle) and lip dark red, obovate-sagittate with laciniate margins (vs. tan with white, oblong, with entire margins) and apex rounded (vs. obtuse-retuse). (Fig. 5, Fig. 6).

**Additional *Scaphosepalum* species examined**: *Scaphosepalum breve*: ECUADOR. Zamora Chinchipe: San Antonio, por una quebrada, 1140 m alt., 30 April 2011, *M.M. Jiménez & M. Jiménez Villalta* 19 (LOJA!: 26652); Cordillera Del Cóndor, between Paquisha and Mayaicu, cultivated by Ecuagenera, Gualaceo, 27 July 2004, *A. Hirtz* 8918 (SEL!: 057536); Steep hills near Zamora, 1275 m alt., 21 May 2022, *M.M. Jiménez & M. Jiménez Villalta* 1380 (HUTPL!: 14735).

*Scaphosepalum ovulare*: ECUADOR. Zamora Chinchipe: Cerca de La Pituca, 1383 m alt., 3 October 2020, *M.M. Jiménez & M. Jiménez Villalta* 963 (HUTPL!: 14721).

*Scaphosepalum rapax*: ECUADOR. Zamora Chinchipe: Cordillera del Cóndor, epiphytic in cloud forest east of Paquisha, 1200 m alt., 23 January 1992, *C. Luer, J. Luer, P. Jesup, A. Jesup & A. Hirtz* 16128 (SEL!: 057576); Near Guayzimi, 914 m alt., 29 January 2017, *M.M. Jiménez, M. Jiménez Villalta & H. Villalta* 413 (HUTPL!: 14653).

*Scaphosepalum pulvinare*: ECUADOR. Zamora Chinchipe: Rio Calagras, San Juan Bosco, cultivated at the Jardin Botanico de Uzhupud by Father Angel Andreetta, collected by A. Andreetta & M. Portillo, 1500 m alt., 22 July 1985, *C. Dodson & A. Embree* 15956 (MO!: 4526048).

*Scaphosepalum tiaratum*: ECUADOR. Morona Santiago: Cordillera de Huaracayo, east of Cordillera del Cóndor and Río Coangos. Flat-topped summit of Cerro Ijiach Naint, east of Shuar village of Tinkimints, 2000 m alt., 21 March 2001, *L. Jost, D.A. Neill, P.E. Berry & J.M. Manzanares 3122* (MO!: 4526050).

## Supplementary Material

XML Treatment for Scaphosepalum lesterlapoi
